# Incidence and Factors Associated with Postoperative Delayed Hyponatremia after Transsphenoidal Pituitary Surgery: A Meta-Analysis and Systematic Review

**DOI:** 10.1155/2021/6659152

**Published:** 2021-04-10

**Authors:** Cheng-Chi Lee, Yu-Chi Wang, Yu-Tse Liu, Yin-Cheng Huang, Peng-Wei Hsu, Kuo-Chen Wei, Ko-Ting Chen, Ya-Jui Lin, Chi-Cheng Chuang

**Affiliations:** ^1^Department of Neurosurgery, Chang Gung Memorial Hospital at Linkou and Chang Gung University, Taoyuan 333, Taiwan; ^2^Department of Biomedical Engineering, National Taiwan University, Taipei 10617, Taiwan; ^3^Graduate Institute of Clinical Medical Sciences, Chang Gung University, Taoyuan 333, Taiwan

## Abstract

**Introduction:**

Postoperative delayed hyponatremia is a complication associated with transsphenoidal pituitary surgery. Due to a wide spectrum of symptoms, the reported incidence and predictors of postoperative delayed hyponatremia vary among studies, and this deserves to be reviewed systematically.

**Methods:**

PubMed, EMBASE, and CENTRAL databases were searched until September 1, 2020. Studies were included when (1) the event number of delayed hyponatremia after transsphenoidal pituitary surgery was reported, or (2) the associated factors of such complication were evaluated.

**Results:**

A total of 27 studies were included for meta-analysis. The pooled incidence of overall and symptomatic delayed hyponatremia was 10.5% (95% confidence interval (CI) = 7.4–14.7%) and 5.0% (95% CI = 3.6–6.9%), respectively. No overt variations of the pooled estimates were observed upon subgroups stratified by endoscopic and microscopic procedure, publication year, and patients' age. In addition, 44.3% (95% CI = 29.6–60.2%) of unplanned hospital readmissions within 30 days were caused by delayed hyponatremia. Among the predictors evaluated, older age was the only significant factor associated with increased delayed hyponatremia (odds ratio = 1.16, 95% CI = 1.04–1.29, *P* = 0.006).

**Conclusion:**

This meta-analysis and systematic review evaluated the incidence of postoperative delayed hyponatremia and found it as a major cause of unplanned readmissions after transsphenoidal pituitary surgery. Older patients are more prone to such complications and should be carefully followed. The retrospective nature and heterogeneity among the included studies and the small number of studies used for risk factor evaluation might weaken the corresponding results. Future prospective clinical studies are required to compensate for these limitations.

## 1. Introduction

Postoperative delayed hyponatremia, defined as serum sodium <130–135 mmol/L, can occur in 1.8–35% of patients who underwent transsphenoidal pituitary tumour surgery, with the peak incidence reported on day 7-8 following surgery [1–3]. Although the majority of patients with delayed hyponatremia are asymptomatic and recover spontaneously [4], patients whose serum sodium levels continue to drop below 125 mmol/L may become symptomatic [5]. Patients with hyponatremia may experience symptoms including headache, nausea, vomiting, lethargy, and confusion and can suffer from seizures, coma, and even death if left untreated in severe cases [1, 2, 6]. In particular, a serum sodium level of lower than 120 mmol/L was shown to associate with a 59.7% 5-year mortality [7]. Postoperative delayed hyponatremia was reported to be a common cause for unexpected hospital readmissions after transsphenoidal pituitary tumour surgery [2, 8], and thus preventive measures have been implemented to reduce these events [9–11]. As such, identifying patients with increased risk of developing delayed hyponatremia is critical to the postoperative care of transsphenoidal pituitary surgery [12–14].

Several pathophysiological mechanisms of posttranssphenoidal pituitary surgery delayed hyponatremia have been proposed, with the syndrome of inappropriate antidiuretic hormone (SIADH) and cerebral salt wasting (CSW) syndrome as the main stream models [1–3]. Partial and complete disruption in the hypothalamic-pituitary gland regulation after surgery can lead to an overproduction of antidiuretic hormone and hence hyponatremia observed in SIADH. On the other hand, perturbed circulating natriuretic peptide levels and decreased sympathetic stimulation to the kidney associated with CSW are rare but potential causes of postoperative delayed hyponatremia in patients undergoing transsphenoidal pituitary surgery [1–3]. Association of patient sex, age, tumour size, and type of surgery with postoperative delayed hyponatremia has been illustrated previously [1–3]. Due to the spectrum of symptoms, disperse awareness of the complication, different aetiology, and the postoperative management protocols, the reported incidence and severity of delayed hyponatremia after transsphenoidal pituitary surgery vary widely and deserve to be scrutinized systematically. Furthermore, a detailed review on the risk factors for postoperative delayed hyponatremia is also warranted.

The aims of this systematic review and meta-analysis are to address (1) the incidence of postoperative delayed hyponatremia following transsphenoidal pituitary surgery and its role on unplanned readmission and (2) the potential predictors of such complication by reviewing the published literature.

## 2. Methods

### 2.1. Search Strategy and Selection Criteria

A literature search through the major public databases (i.e., PubMed, EMBASE, and CENTRAL) was conducted using “transsphenoidal,” “pituitary neoplasms,” “pituitary adenoma,” “surgery,” and “hyponatremia” as keywords combined with Boolean operators and using Medical Subject-Headings (MeSH) terms where appropriate for studies published prior to September 1, 2020. As an example, the search string used for PubMed was

(“pituitary neoplasms/surgery” [MeSH Major Topic] OR “transsphenoidal” [All Fields]) AND hyponatremia [MeSH].

The inclusion criteria were studies that either reported (1) event number and/or incidence of delayed hyponatremia during the postoperative follow-up of either endoscopic or microscopic transsphenoidal surgery for pituitary tumours or (2) the associated factors of such delayed hyponatremia. In contrast, studies where (1) early or delayed onset hyponatremias cannot be clearly distinguished, (2) paediatric patients were enrolled only, (3) no quantitative outcomes of interest were available, and (4) language other than English was adopted were excluded from the present review. Regarding publication type, only cohort studies performed prospectively or retrospectively were eligible for inclusion, while letters, commentaries, editorials, proceedings, case reports, and personal communications were not considered. In addition, the reference lists of included studies were hand-searched to identify other potentially relevant studies. This systematic review and meta-analysis was conducted in accordance with the Preferred Reporting Items for Systematic Reviews and Meta-Analyses (PRISMA) guidelines [15].

### 2.2. Main Outcome Measures and Data Extraction

#### 2.2.1. The Outcomes of Interest Included

1. The incidence of postoperative delayed hyponatremia, defined as serum sodium concentration <130–135 mmol/L occurring on a postoperative day (POD) three and onwards
2. The incidence of symptomatic delayed hyponatremia, defined as delayed hyponatremia with patient-reported symptoms
3. The rate of postoperative delayed hyponatremia in unplanned hospital readmission within 30 days after surgery
4. The demographic and/or clinical factors associated with the occurrence of delayed hyponatremias

The eligibility of studies identified via the above search and selection strategy was confirmed by two independent reviewers (CCL and YCH), and a third reviewer (CCC) was consulted where there was uncertainty regarding eligibility. From these eligible studies and when available, the following information was extracted: the name of the first author, year of publication, study design, study country, the total number of patients, type of surgery, patients' mean age, sex, tumour characteristics (i.e., functioning or nonfunctioning; macro- or microadenomas), aetiology of hyponatremia, and main outcomes of interest. Due to the nature of the present review, raw patient data and private information were neither required nor used, and thus informed consent from study subjects or approval from the institutional review board was waived.

### 2.3. Quality Assessment

We assessed the quality of included studies using the Newcastle-Ottawa scale (NOS) for cohort studies as recommended by the Cochrane Nonrandomized Studies Methods Working Group [16]. This scale awards a maximum of nine points to each study representing four points for the adequate selection of cohort participants, two points for the comparability of cohort participants on the basis of the design and analysis, and three points for the adequate ascertainment of outcomes. Quality assessment was performed by two independent reviewers (YCW and YTL), and a third reviewer (PWH) was consulted if any uncertainties occurred.

### 2.4. Statistical Analysis

Event rates of the outcomes of interest with 95% confidence interval (CI) were extracted or calculated from each individual study, and then the summary effect with 95% CI was estimated. Planned subgroup analyses of overall postoperative delayed hyponatremia incidence were performed based on the type of surgery, publication year, and patients' mean age. For the associated factors, the OR with 95% CI was extracted from the included studies, and pooled estimates were generated.

An *χ*^2^-based test of homogeneity was performed to determine the inconsistency index (*I*^2^) and *Q* statistics. If the *I*^2^ statistic was >50%, the random-effects model was utilized to calculate pooled effects, and a fixed-effect model was employed when otherwise. A 2-sided *P* value of <0.05 was considered to indicate the statistical significance of the pooled estimate. Sensitivity analysis for the endpoints was addressed by the leave-one-out approach. Potential publication bias was assessed by Egger's test (whenever the total number of evaluated studies was >10) [17], respectively. The absence of publication bias was indicated by the data points forming a symmetric funnel-shaped distribution and a corresponding one-tailed *P* > 0.05. All analyses were performed using the Comprehensive Meta-Analysis statistical software, version 2.0 (Biostat, Englewood, NJ, USA).

## 3. Results

### 3.1. Characteristics of Included Studies and Basic Patient Demographics

**Figure 1:**
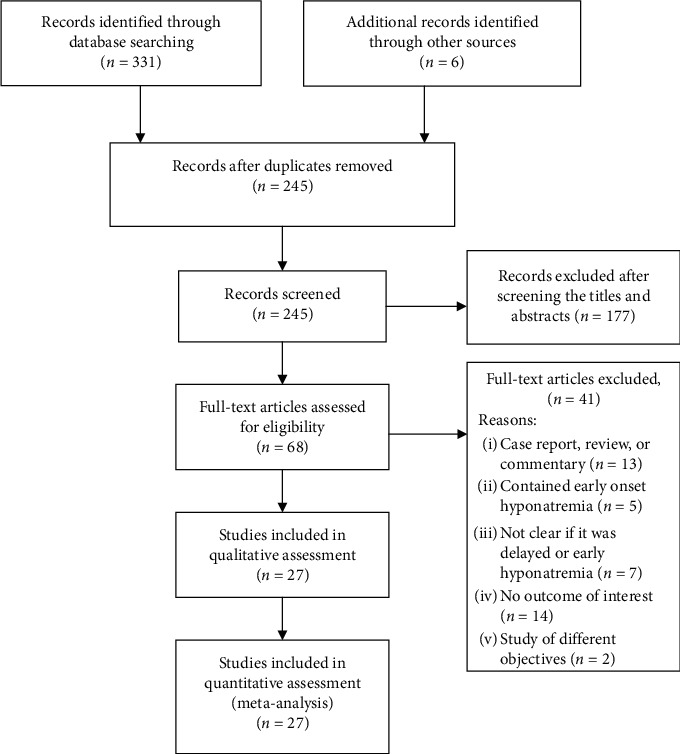
PRISMA flow diagram of study selection. The number of search hits corresponding to each step of the systematic literature search, qualitative review, and quantitative analysis is shown. The reasons for search hit exclusion are described.

The electronic search and study selection process is shown in Figure 1. After excluding duplicates, the search yielded a total of 245 unique citations, from which 68 candidate studies were identified after screening titles and abstracts electronically. Among the candidate studies that underwent full-text assessment, 41 were excluded for mixed or indistinguishable results of early from delayed onset hyponatremias, not reporting outcomes of interest, of different study objectives, and being a case report, review, or commentary (Figure 1). Finally, 27 studies met the eligibility criteria and were included for meta-analysis [4, 6, 9–13, 18–37].

Specifically, 10 of the 27 studies were published before 2011 [4, 21, 22, 25, 26, 28, 31, 32, 34, 37], with the remaining studies published later [6, 9–13, 18–20, 23, 24, 27, 29, 30, 33, 35, 36]; 3 of the 27 studies were prospective studies, whereas the others were conducted retrospectively [4, 6, 9–13, 18–20, 22, 23, 25–30, 32–37] (Table 1). The study size of the 27 included studies was between 84 and 2297 patients, encompassing a total of 11,356 patients. The mean age of patients ranged from 42 to 54 years, and the proportion of males was 32%–63%. The pathological tumour types varied among studies, with nonfunctioning pituitary adenoma as the major type. While 3 studies exclusively reported follow-up of patients with macroadenoma [6, 30, 33], 12 studies collected data from mixed patient cohorts diagnosed with macroadenoma or microadenoma [4, 11, 21–23, 25, 27, 28, 31, 32, 35, 37], and the remaining studies did not provide such information [9, 10, 12, 13, 18–20, 24, 26, 29, 34, 36]. While 6 and 10 studies recruited patients receiving microscopic [22, 25, 26, 28, 31, 34] and endoscopic transsphenoidal procedure [4, 12, 13, 21, 23, 29, 30, 33, 36, 37], respectively, the majority of studies did not specifically categorize patients into types of transsphenoidal surgery [6, 9–11, 18–20, 24, 27, 32, 35].

In general, the definition used for postoperative delayed hyponatremia was comparable among studies, with serum sodium of <130–135 mmol/L as the cut-off and onset on or beyond postoperative day 3 (Table 2). The incidence of overall delayed hyponatremia and those with symptoms in the individual study was in the range of 2–35% and 2.1–19.8%, respectively. Among the 6 studies that reported delayed hyponatremia as a cause of unplanned hospital readmission within 30 days after the initial transsphenoidal surgery, the rate of delayed hyponatremia ranged from 21% to 61% [11, 20, 27, 29, 30, 36].

### 3.2. Incidence of Overall and Symptomatic Postoperative Delayed Hyponatremia

**Figure 2:**
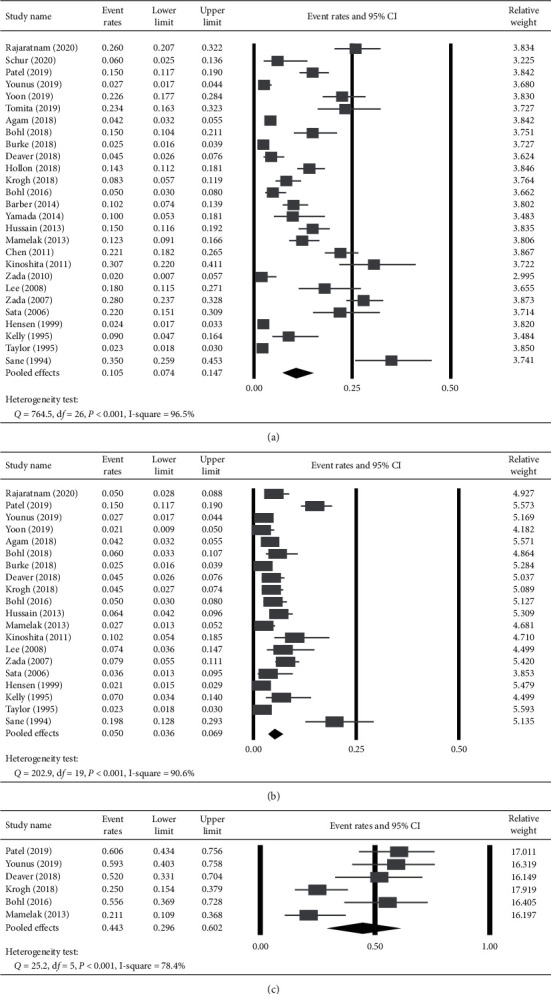
Main results of meta-analysis: incidence and hospital readmissions. The values of individual studies and pooled estimates of (a) overall incidence of delayed hyponatremia, (b) incidence of symptomatic delayed hyponatremia, and (c) rate of hospital readmission within 30-day postoperatively due to delayed hyponatremia are shown. A random-effects model was adopted based on the results from the heterogeneity test.

High heterogeneity in the reported incidence of delayed hyponatremia was found across the 27 studies (*Q* statistic = 764.5, *I*^2^ = 96.5%, *P* < 0.001); therefore, the random-effects model was used. Consequently, the pooled overall incidence of postoperative delayed hyponatremia was 10.5% (95% CI = 7.4–14.7%) (Figure 2(a)). Twenty of 27 studies specifically reported incidence of symptomatic delayed hyponatremia after transsphenoidal pituitary surgery [4, 6, 9–11, 13, 18, 20, 22, 24–32, 34, 36], and a high heterogeneity was observed across studies (*Q* statistic = 202.9, *I*^2^ = 90.6%, *P* < 0.001, Figure 2(b)). Based on the random-effects model, the pooled incidence of symptomatic delayed hyponatremia was 5% (95% CI = 3.6–6.9%).

### 3.3. Subgroup Analyses of Overall Postoperative Delayed Hyponatremia Incidence

Table 3 summarizes pooled estimates for the incidence of postoperative delayed hyponatremia stratified by type of surgery, publication year, patients' mean age, and the criteria of delayed hyponatremia. Again, the heterogeneity in all subgroups was high (all *I*^2^ > 75%). The pooled incidence of delayed hyponatremia following endoscopic transsphenoidal surgery was 11.3% (95% CI = 7.5–16.8%) and 9.2% (95% CI = 3.6–21.8%) in microscopic transsphenoidal surgery. The pooled postoperative delayed hyponatremia incidence of studies published before 2011 and after 2011 was 12.1% (95% CI = 5.6–24.1%) and 9.7% (95% CI = 6.8–13.5%), respectively. The pooled postoperative delayed hyponatremia incidence of studies with a patient cohort aged below 50 years in average and that of studies ≥50 years were similar (<50 years: 11.7% [95% CI = 7.1–18.5%]; ≥50 years: 12.1% [95% CI = 8.5–16.8%]). The pooled postoperative delayed hyponatremia incidence of studies using the definition for hyponatremia of <135 mmol/L and others was similar to that of the studies using other definitions (<135 mmol/L: 10.4% [95% CI = 7.0–15.1%]; others: 11.0% [95% CI = 4.5–24.4%]).

### 3.4. The Rate of Postoperative Delayed Hyponatremia in Unplanned Readmission within 30 Days

Among the 6 studies that reported the rate of delayed hyponatremia as a cause of unplanned readmission within 30 days following transsphenoidal pituitary surgery [11, 20, 27, 29, 30, 36], high heterogeneity was found (*Q* statistic = 25.2, *I*^2^ = 78.4%, *P* < 0.001, Figure 2(c)). The pooled estimates revealed that delayed hyponatremia accounted for 44.3% (95% CI = 29.6–60.2%) of all readmissions within 30 days.

### 3.5. Sensitivity Analysis and Publication Bias

**Figure 3:**
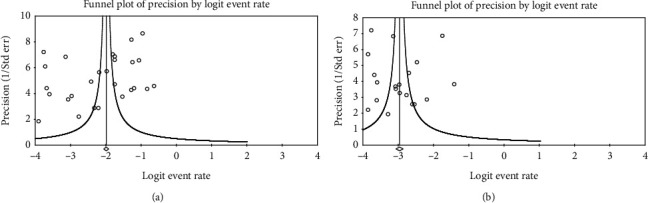
Funnel plot for verification of publication bias in the present meta-analysis. Egger's test was utilized to verify the presence of publication bias in the meta-analysis for (a) overall incidence of delayed hyponatremia and (b) incidence of symptomatic delayed hyponatremia.

Sensitivity analyses were performed using the leave-one-out approach in which meta-analysis was performed with each study removed in turn (Table 4). The summary effects of overall incidence of postoperative delayed hyponatremia, symptomatic delayed hyponatremia, and readmission due to delayed hyponatremia remained between 10 and 11%, unchanged (5%), and 41–49%, respectively, indicating that the meta-analysis had good reliability and the data was not overly influenced by each study.  Figure 3 shows that there was no publication bias in the findings with regard to the incidence of overall delayed hyponatremia (*t* = 1.106, *P* = 0.140, Figure 3(a)) and symptomatic delayed hyponatremia (*t* = 0.153, *P* = 0.440, Figure 3(b)) as exemplified by Egger's test. The rate of readmissions resulting from postoperative delayed hyponatremia was not subjected to Egger's test due to the limited number of studies included (i.e., 6 in total).

### 3.6. Risk Factors of Postoperative Delayed Hyponatremia

**Figure 4:**
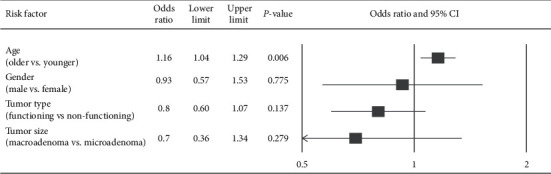
Meta-analysis for the associations between delayed hyponatremia and age, gender, tumour type, and tumour size.

Meta-analyses were performed to determine the associations between postoperative delayed hyponatremia and patients' characteristics including age [6, 12, 13, 23, 26–28], sex [4, 6, 12, 24–28, 30–32], tumour type (functioning vs. nonfunctioning) [12, 13, 22, 24, 26–28, 31, 32], and tumour size (macroadenomas vs. microadenomas) [4, 25, 27, 28, 31, 32], for these factors were the most consistently reported across the included studies. The results revealed that older age (over 55–60 years) was significantly associated with an increased likelihood of incident postoperative delayed hyponatremia, with a pooled OR of 1.16 (95% CI = 1.04 to 1.29, *P* = 0.006), whereas no significant associations were found with respect to sex, tumour type, or size (Figure 4).

### 3.7. Quality Assessment

The quality rating of the individual study is shown in Table 1. The total score ranged from 6 to 8 with an average of 6.3, suggesting that the studies included were of moderate quality (Table 1).

## 4. Discussion

The present study is the most updated systematic review and the first in reporting the results of a corresponding meta-analysis on the incidence and risk factors of postoperative delayed hyponatremia after transsphenoidal pituitary surgery. We found that the pooled incidences of postoperative delayed hyponatremia overall and those with symptoms were 10 and 5%, respectively. Delayed hyponatremia contributed to 44% of the unplanned hospital readmissions within 30 days. Significant heterogeneity existed among the studies, whereas these estimates were robust under sensitivity analyses. Among different subgroups categorized by surgical type, mean age, and publication year, the incidences seemed not to vary greatly from the main findings. Older age was associated with a slightly increased risk of postoperative delayed hyponatremia, while male gender, tumour type, and size showed no significant associations.

Previously, Cote et al. (2016) have systematically reviewed the incidence and predictors of delayed hyponatremia after transsphenoidal pituitary surgery based on 10 studies with a total of 2,947 patients [14] which were all included in our meta-analysis. The authors have focused on symptomatic delayed hyponatremia only and reported an event rate of about 4–12%, with a variety of potential predictors proposed. However, no quantitative estimations were conducted. An American registry-based study surveyed the postoperative complications in 1,240 patients who underwent transsphenoidal pituitary surgery during 2006–2015 and found hyponatremia as the major cause for unplanned rehospitalizations, representing 29.5% of such readmissions [8].

Among the studies evaluating predictors of delayed hyponatremia after transsphenoidal pituitary surgery, most studies found that older age was associated with the occurrence of delayed hyponatremia [12, 13, 23, 26–28]. Despite the numerous reports testing the predictive values of females, macroadenoma, and a diagnosis of Cushing's disease in delayed hyponatremia [4, 6, 12, 13, 22, 24–28, 30–32], the result of the present meta-analysis indicated that older age was the only significant associated factor. The studies that observed significant associations between sex, tumour size, or tumour pathology and postoperative delayed hyponatremia were mostly published prior to 2013 [4, 22, 24, 26, 31], where potential differences in the awareness of the complication and postoperative management approach from the study afterwards may exist and thus led to inconsistency.

SIADH, which commonly results from iatrogenic injury of the neurohypophysis during surgical exploration and leads to degeneration of magnocellular neurons and excessive release of antidiuretic hormone, is proposed as an aetiology of postoperative delayed hyponatremia [1, 2]. Another less commonly accepted explanation for such complication is CWS secondary to increased release of atrial and cerebral natriuretic peptide. Other potential causes of postoperative serum sodium perturbation include adrenocortical insufficiency, hypothyroidism, hypernatremia overcorrection, and volume overload. Although our original aim was to evaluate the pooled rates of delayed hyponatremia due to different aetiologies, this is precluded by the limited studies that reported definite aetiologies in the literature.

To date, there are only limited studies that directly compared the potential impact of endoscopic versus microscopic transsphenoidal pituitary surgery on the occurrence of delayed hyponatremia. It may be intuitive to speculate that less iatrogenic injury in the neurohypophysis is associated with endoscopic than microscopic procedures due to improved visualization. However, a prior systematic review compared postoperative complications between these two surgical approaches and only found 3 published articles reporting relevant data [38], with no significant differences for the SIADH between endoscopic and microscopic procedures. The present and a separate systematic review [14] arrived at similar conclusions in that the postoperative delayed hyponatremia rate did not differ with regard to endoscopic versus microscopic procedures. Future well-designed randomized controlled trials are warranted to confirm this finding.

Due to the increased awareness of postoperative delayed hyponatremia and the evidence showing such complication as the main cause of unplanned readmissions after transsphenoidal pituitary surgery, clinicians have devoted to constructing and implementing outpatient care algorithm in preventing delayed hyponatremia-associated rehospitalizations [12–14]. In brief, the preventive measures involved a fluid restriction (to 1.0–1.5 L/day) that was mandatory or guided by an active screening of serum sodium levels 5–7 days after discharge from the hospital. Based on these reports, mandatory fluid restriction starting from the day of discharge from the hospital was vital in preventing the occurrence of symptomatic hyponatremia that resulted in readmission [10, 11], whereas active screening-guided fluid intake control was not sufficient in this respect [9]. These findings perhaps reflect the fact that SIADH is the more prevalent aetiology of delayed hyponatremia after transsphenoidal pituitary surgery than CSW, where hyper- and hypovolemia contribute to the pathophysiology of the two respective conditions [2].

As with previously published studies of similar nature, the interpretation of the results of the present meta-analysis is limited by the heterogeneity across the eligible studies, although they were selected based on stringent criteria and a thorough literature search. To address this issue, we have adopted the random-effects model, study stratification, and sensitivity analysis. Nonetheless, no specific source of heterogeneity could be indicated. Additionally, the retrospective nature of most included studies presents as a potential source of selection bias overall, whereas the small number of studies used for risk factor evaluation may weaken the corresponding results. In the present study, all functional adenomas were pooled together, and we were not able to look at Cushing's disease and acromegaly separately because most of the included studies did not separately report the outcomes by tumour pathologies. Only a rough categorization of age as a risk factor (above 50 vs. below 50) was done in the present analysis due to the nature of data extracted. More research efforts and prospective clinical studies in the field are required to compensate for the limitations discussed herein, especially with an in-depth investigation on the risk factor age.

## 5. Conclusion

Taking together, this systematic review and meta-analysis demonstrated an incidence of hyponatremia which is unignorable following transsphenoidal pituitary surgery. Furthermore, delayed hyponatremia is a major cause of 30-day readmissions in these patients. Among the potential risk factors, only old age is associated with this complication. Despite several limitations, the present findings may enhance the surgeons' awareness of the patient profile with an increased risk of delayed hyponatremia. Based on such understanding, clinicians may be motivated to develop preventive or interventive measures to reduce the possibility and impact of postoperative delayed hyponatremias.

## Figures and Tables

**Table 1:** Summary of demographics of selected studies.

First author (year)	Study design	Country	No. of pts	Type of surgery	Male (%)	Age (year)	Tumour pathology	Macroadenoma vs. Microadenoma	NOS score
Rajaratnam (2020)	Retrospective	India	222	mTSS/eTSS	63	45	NFPA 100%	100% vs. 0%	6
Schur (2020)	Retrospective	Canada	84	eTSS	56	54.1	N/A	100% vs. 0%	6
Patel (2019)	Retrospective	USA	367	eTSS	46	48.5	NFPA 44%, PRL 13%, ACTH-secreting 13%, GH-secreting 4%, atypical 1%, RCC 10%, craniopharyngioma 4%, meningioma 3%, others 3%	100% vs. 0%	6
Younus (2019)	Retrospective	USA	584	eTSS	51	N/A	NFPA 39%, acromegaly 21%, PRL 26%, Cushing 14%	N/A	8
Yoon (2019)	Retrospective	Korea	234	eTSS	49	54.4	NFPA 100%	N/A	8
Tomita (2019)	Retrospective	Japan	107	eTSS	36	54	NFPA 69%, GHoma 22%, Cushing 5%, PRLoma 4%, TSHoma 1%	N/A	6
Agam (2018)	Retrospective	USA	1153	mTSS/eTSS	46	49.5	NFPA 54.0%, GHoma 14.6%, PRLoma 14.1%, ACTHoma 12.2%, other 5.0%	N/A	6
Bohl (2018)	Retrospective	USA	172	mTSS/eTSS	57	51.9	NFPA 59.6%, PRLoma 14.9%, acromegaly 8.5%, Cushing 9.0%, TSHoma 0.5%, others 8.0%	N/A	6
Burke (2018)	Retrospective	USA	788	mTSS/eTSS	45	47.7	NFPA 20.3%, PRLoma 6.6%, Cushing 10.5%, acromegaly 12.3%, TSHoma 0.5%, RCC 8.9%, other 4.7%	N/A	6
Deaver (2018)	Retrospective	USA	287	mTSS/eTSS	51	53	NFPA: 15.7%, functioning: 82.9%, other: 1.4%	85.7% vs. 14.3%	6
Hollon (2018)	Retrospective	USA	400	eTSS	54	53.9	NF 59.8%, acromegaly 22.8%, Cushing 13.0%, PRL 4.0%, TSHoma 0.5%	84.7% vs. 15.3%	8
Krogh (2018)	Retrospective	UK	314	mTSS/eTSS	49	53.1	NFPA 40.8%, acromegaly 12.4%, Cushing 4.4%, PRL 4.5%, craniopharyngioma 6.4%, Rathke cleft cyst 6.1%, meningioma 3.2%, other 19.4%	41% vs. 59%	8
Bohl (2016)	Retrospective	USA	303	mTSS/eTSS	54	52.9	NFPA 67.3%, acromegaly 9.9%, Cushing 7.9%, TSHoma 1.0%, PRLoma 11.9%, others 2%	N/A	6
Barber (2014)	Retrospective	USA	344	mTSS/eTSS	45	48	NFPA 66.3%, functional adenoma 16.0%, RCC 14.0%	N/A	6
Yamada (2014)	Retrospective	Japan	90	mTSS/eTSS	48	42	TSHoma 100%	82% vs.18%	6
Hussain (2013)	Prospective	USA	339	mTSS/eTSS	39	48	NFPA 33%, Cushing 24%, PRLoma 10.5%, acromegaly 8.5%, RCC 2.9%	N/A	6
Mamelak (2013)	Retrospective	USA	300	eTSS	43	51.6	NF 50.7%, ACTHoma 4%, GHoma 13.7%, PRLoma 5.7%, RCC 10%, other 15.9%	N/A	6
Chen (2011)	Prospective	China	385	eTSS	53	51	NFPA 100%	8% vs. 92%	6
Kinoshita (2011)	Retrospective	Japan	88	mTSS	32	47.9	NFPA 27%, GHoma 20.5%, PRLoma35.2%, FSH or LHoma 13.6%, TSHoma 3.4%	N/A	6
Zada (2010)	Retrospective	USA	169	eTSS	54	44	Acromegaly 100%	61% vs. 39%	6
Lee (2008)	Retrospective	Korea	94	mTSS	54	42.8	NFPA 53%, PRLoma 31%, GHoma 12%, ACTHoma 4%	10% vs. 90%	6
Zada (2007)	Retrospective	USA	369	eTSS	46	48	NFPA 50%, PRLoma 12%, GHoma 12%, Cushing 9%, RCC 9%, others 8%	76% vs. 24%	6
Sata (2006)	Retrospective	Japan	105	mTSS/eTSS	35	43	NFPA 38%, acromegaly 25%, RCC 15%, PRLoma 12%, Cushing 2%, others 8%	11% vs. 89%	6
Hensen (1999)	Retrospective	Germany	1571	mTSS	43	44.5	NFPA 34%, Cushing 15%, acromegaly 26%, PRLoma 22.8%, others 2.2%	64% vs. 30%	6
Kelly (1995)	Retrospective	USA	99	mTSS	32	45	N/A	35% vs. 64%	6
Taylor (1995)	Retrospective	USA	2297	mTSS	36	N/A	N/A	N/A	6
Sane (1994)	Prospective	Finland	91	mTSS	48	45	NFPA 29%, GHoma 26%, ACTHoma 20%, PRLoma 15%, gonadotropinomas 3%, others 7%	35% vs. 65%	6

ACTHoma, adrenocorticotropic hormone-secreting adenoma; eTSS, endoscopic transsphenoidal surgery; FSH, follicular-stimulating hormone; GHoma, growth hormone-secreting adenoma; LHoma, luteinizing hormone adenoma; mTSS, microscopic transsphenoidal surgery; N/A, not applicable; NFPA, nonfunctioning pituitary adenoma; NOS, Newcastle-Ottawa Scale; PRLoma, prolactinoma; RCC, Rathke's cleft cysts; TSHoma, thyroid-stimulating hormone-secreting adenoma.

**Table 2:** The summary of outcomes.

First author (year)	No of patient	Criteria of delayed hyponatremia	Delayed hyponatremia, *N* (%)	Symptomatic delayed hyponatremia, *N* (%)	As cause for unplanned readmission within 30 days
Rajaratnam (2020)	222	Serum sodium <135 mmol/L on or after POD 3	58 (26%)	11 (5%)	N/A
Schur (2020)	84	Serum sodium <135 mmol/L, on or after POD 5	5 (6%)	N/A	N/A
Patel (2019)	367	Serum sodium <135 mmol/L with associated symptoms	55 (15%)	55 (15%)	20/33
Younus (2019)	584	Serum sodium <135 mmol/L under routine screening on POD 6, with associated symptoms	16 (2.7%)	16 (2.7%)	16/27
Yoon (2019)	234	Serum sodium <135 mmol/L on or after POD 3	53 (22.6%)	5 (2.1%)	N/A
Tomita (2019)	107	Serum sodium <135 mmol/L on or after POD 3	25 (23.4%)	N/A	N/A
Agam (2018)	1153	Serum sodium <135 mmol/L with associated symptoms	48 (4.2%)	48 (4.2%)	N/A
Bohl (2018)	172	Serum sodium <135 mmol/L under screening	29 (15%)	11 (6%)	N/A
Burke (2018)	788	Serum sodium <135 mmol/L with associated symptoms	20 (2.5%)	20 (2.5%)	N/A
Deaver (2018)	287	Serum sodium <135 mmol/L with associated symptoms	13 (4.5%)	13 (4.5%)	13/25
Hollon (2018)	400	Serum sodium <135 mmol/L on or after POD 3	54 (14.3%)	N/A	N/A
Krogh (2018)	314	Serum sodium <130 mmol/L on POD 6–8	26 (8.3%)	14 (4.46%)	14/56
Bohl (2016)	303	Serum sodium <135 mmol/L with associated symptoms	15 (4.95%)	15 (4.95%)	15/27
Barber (2014)	344	Serum sodium <135 mmol/L on or after POD 3	35 (10.2%)	N/A	N/A
Yamada (2014)	90	Serum sodium <135 mmol/L on or after POD 3	9 (10%)	N/A	N/A
Hussain (2013)	339	Serum sodium <130 mmol/L on POD 6–13	50 (15%)	22 (6.4%)	N/A
Mamelak (2013)	300	Serum sodium <133 mmol/L on or after POD 3	37 (12.33%)	8 (2.67%)	8/38
Chen (2011)	385	Serum sodium <135 mmol/L on or after POD 3	85 (22.1%)	N/A	N/A
Kinoshita (2011)	88	Serum sodium <135 mmol/L with associated symptoms	27 (30.7%)	9 (10.2%)	N/A
Zada (2010)	169	Serum sodium <135 mmol/L on or after POD 3	4 (2%)	N/A	N/A
Lee (2008)	94	Serum sodium <135 mmol/L on or after POD 3	17 (18%)	7 (7.4%)	N/A
Zada (2007)	369	Serum sodium <135 mmol/L on or after POD 3	103 (28%)	29 (7.9%)	N/A
Sata (2006)	105	Serum sodium <135 mmol/L on or after POD 3	24 (22%)	4 (3.64%)	N/A
Hensen (1999)	1571	Serum sodium <132 mmol/L on or after POD 3	37 (2.4%)	32 (2.1%)	N/A
Kelly (1995)	99	Serum sodium <135 mmol/L on or after POD 3	9 (9%)	7 (7%)	N/A
Taylor (1995)	2297	Serum sodium <135 mmol/L with associated symptoms	53 (2.3%)	53 (2.3%)	N/A
Sane (1994)	91	Serum sodium <132 mmol/L on or after POD 3	32 (35%)	18 (19.8%)	N/A

POD, postoperative day; N/A, not applicable.

**Table 3:** Stratified meta-analysis of the incidence of delayed hyponatremia.

	Number of studies	*Q* statistics	*I*-square (%)	Pooled event rate with 95% CI
Type of surgery				
eTSS	11	164.8	93.9	0.113 (0.075, 0.168)
mTSS	7	280.2	97.7	0.092 (0.036, 0.218)

Publication year				
Before 2011	10	471.3	98.1	0.121 (0.056, 0.241)
2011+	17	269.6	94.1	0.097 (0.068, 0.135)

Mean age				
<50 years	15	433.9	96.8	0.117 (0.071, 0.185)
50+ years	10	91.2	90.1	0.121 (0.085, 0.168)

Criteria of delayed hyponatremia				
Sodium <135 mmol/L	22	590.4	96.4	0.104 (0.070, 0.151)
Others	5	147.3	97.3	0.110 (0.045, 0.244)

eTSS, endoscopic transsphenoidal surgery; mTSS, microscopic transsphenoidal surgery.

**Table 4:** A sensitivity analysis.

	Statistics with indicated study removed		
Study name	Event rate	Lower limit	Upper limit
Delayed hyponatremia			
Rajaratnam (2020)	0.101	0.071	0.142
Schur (2020)	0.107	0.075	0.150
Patel (2019)	0.103	0.072	0.147
Younus (2019)	0.110	0.078	0.154
Yoon (2019)	0.102	0.071	0.144
Tomita (2019)	0.102	0.071	0.143
Agam (2018)	0.109	0.077	0.152
Bohl (2018)	0.103	0.072	0.146
Burke (2018)	0.108	0.076	0.152
Deaver (2018)	0.104	0.072	0.147
Hollon (2018)	0.106	0.074	0.150
Krogh (2018)	0.111	0.078	0.154
Bohl (2016)	0.108	0.076	0.152
Barber (2014)	0.105	0.073	0.149
Yamada (2014)	0.105	0.074	0.148
Hussain (2013)	0.103	0.072	0.147
Mamelak (2013)	0.104	0.072	0.148
Chen (2011)	0.102	0.071	0.144
Kinoshita (2011)	0.100	0.070	0.141
Zada (2010)	0.110	0.078	0.154
Lee (2008)	0.103	0.072	0.145
Zada (2007)	0.101	0.071	0.141
Sata (2006)	0.102	0.071	0.144
Hensen (1999)	0.111	0.080	0.153
Kelly (1995)	0.106	0.074	0.149
Taylor (1995)	0.112	0.081	0.152
Sane (1994)	0.100	0.070	0.140

Symptomatic delayed hyponatremia			
Rajaratnam (2020)	0.050	0.035	0.070
Patel (2019)	0.047	0.035	0.062
Younus (2019)	0.052	0.037	0.072
Yoon (2019)	0.052	0.037	0.072
Agam (2018)	0.050	0.035	0.072
Bohl (2018)	0.049	0.035	0.069
Burke (2018)	0.052	0.037	0.073
Deaver (2018)	0.050	0.036	0.071
Krogh (2018)	0.050	0.036	0.071
Bohl (2016)	0.050	0.035	0.070
Hussain (2013)	0.049	0.035	0.069
Mamelak (2013)	0.051	0.037	0.072
Kinoshita (2011)	0.048	0.034	0.067
Lee (2008)	0.049	0.035	0.069
Zada (2007)	0.049	0.034	0.069
Sata (2006)	0.051	0.036	0.071
Hensen (1999)	0.053	0.038	0.073
Kelly (1995)	0.049	0.035	0.069
Taylor (1995)	0.052	0.038	0.072
Sane (1994)	0.046	0.034	0.063

Cause for unplanned readmission within 30-day			
Patel (2019)	0.411	0.256	0.585
Younus (2019)	0.415	0.257	0.593
Deaver (2018)	0.429	0.261	0.616
Krogh (2018)	0.491	0.340	0.644
Bohl (2016)	0.422	0.258	0.606
Mamelak (2013)	0.495	0.344	0.646

## Data Availability

The datasets generated and/or analyzed during the current study are available from the corresponding author on reasonable request.
